# Supplementary material for: New climate models reveal faster and larger increases in Arctic precipitation than previously projected
Source: Nat Commun. 2021 Nov 30;12:6765. doi: 10.1038/s41467-021-27031-y (PMC8633026; doi:10.1038/s41467-021-27031-y)
Supplement: Supplementary file 1 — Supplementary Information [file 41467_2021_27031_MOESM1_ESM.pdf]

Supplementary Information for

New climate models reveal faster and larger increases in Arctic precipitation that previously projected

Michelle R. McCrystall[1*], Julienne Stroeve[1,2,3], Mark Serreze[3], Bruce C. Forbes[4] , James A. Screen[5]

[1]Centre for Earth Observation Science, University of Manitoba, Winnipeg, Canada
[2] Department of Earth Sciences, University College London, London, United Kingdom
[3]National Snow and Ice Data Centre, Cooperative Institute for Research in Environmental Science, University of Colorado Boulder, Boulder, Colorado, USA
[4]Arctic Centre, University of Lapland, Rovaniemi, Finland
[5]College of Engineering, Maths, and Physical Sciences, University of Exeter, Exeter, United Kingdom

*Corresponding Author: Michelle McCrystall, michelle.mccrystall@umanitoba.ca

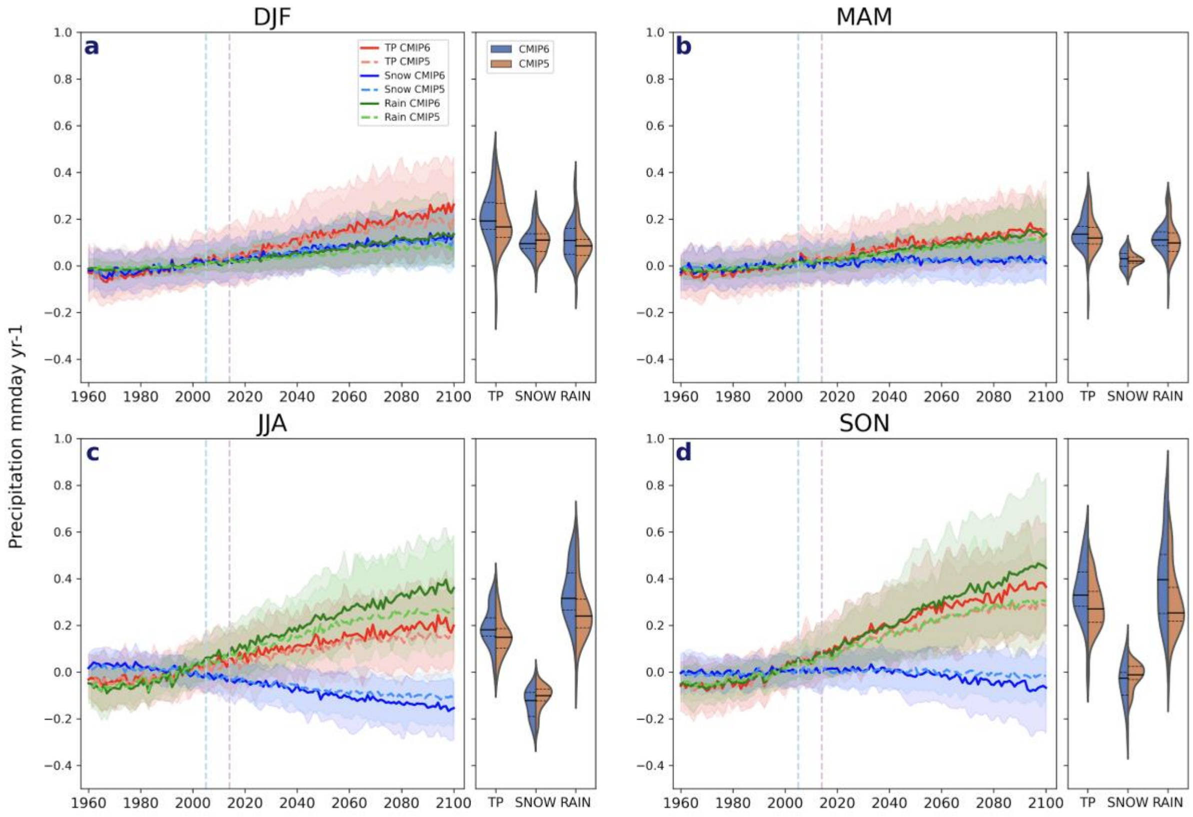

Supplementary Figure 1: Time series of total precipitation, snowfall and rainfall anomalies in CMIP6 and CMIP5 models. Changes in total precipitation (TP) (red, orange), snowfall (snow) (blue, light blue), rainfall (rain) (green, light green) in CMIP6 and CMIP5 relative to the 1981-2009 climatological mean for [a] December-February (DJF), [b] March-May (MAM), [c] June-August (JJA) and [d] September-November (SON). The light blue vertical dashed line represents when the historical period for CMIP5 ends and light purple vertical dashed line represents when the historical period of CMIP6 ends and thereafter the RCP 4.5 and SSP245 climate scenarios for CMIP5 and CMIP6 are used. The shading around each line highlights the spread based upon the lower 5th and 95th percentiles among the model members. The boxplots in each panel represent the model spread in 2100 for each total precipitation (TP), snowfall (snow), and rainfall (rain) with the boxes representing the 25th and 75th percentiles and whiskers indicating the 5th and 95th percentiles. The black vertical line in each represents the mean of all models.

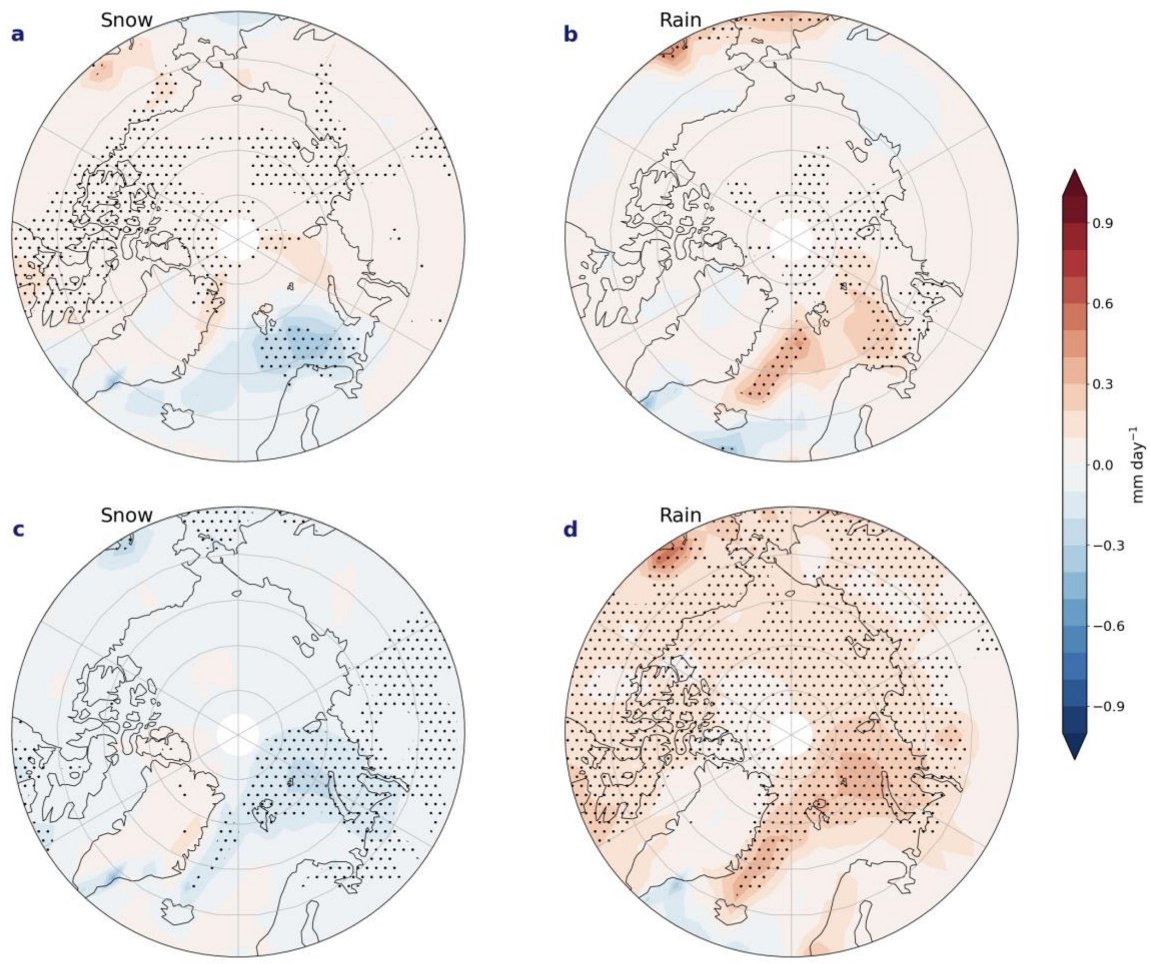

Supplementary Figure 2: Difference in snowfall and rainfall between CMIP6 and CMIP5 by the end of the century. Snowfall change (first column) and rainfall change (second column) at the end of the century (2090-2100) compared to the start of the century (2005-2014) expressed as differences between the CMIP6 multi-model mean and the CMIP5 multi-model mean for SSP2-45 and RCP4.5 scenarios respectively. Hatching indicates statistical significance at 95% confidence level. Results are provided for December-February (DJF) (top row) and September-November (SON) (second row).

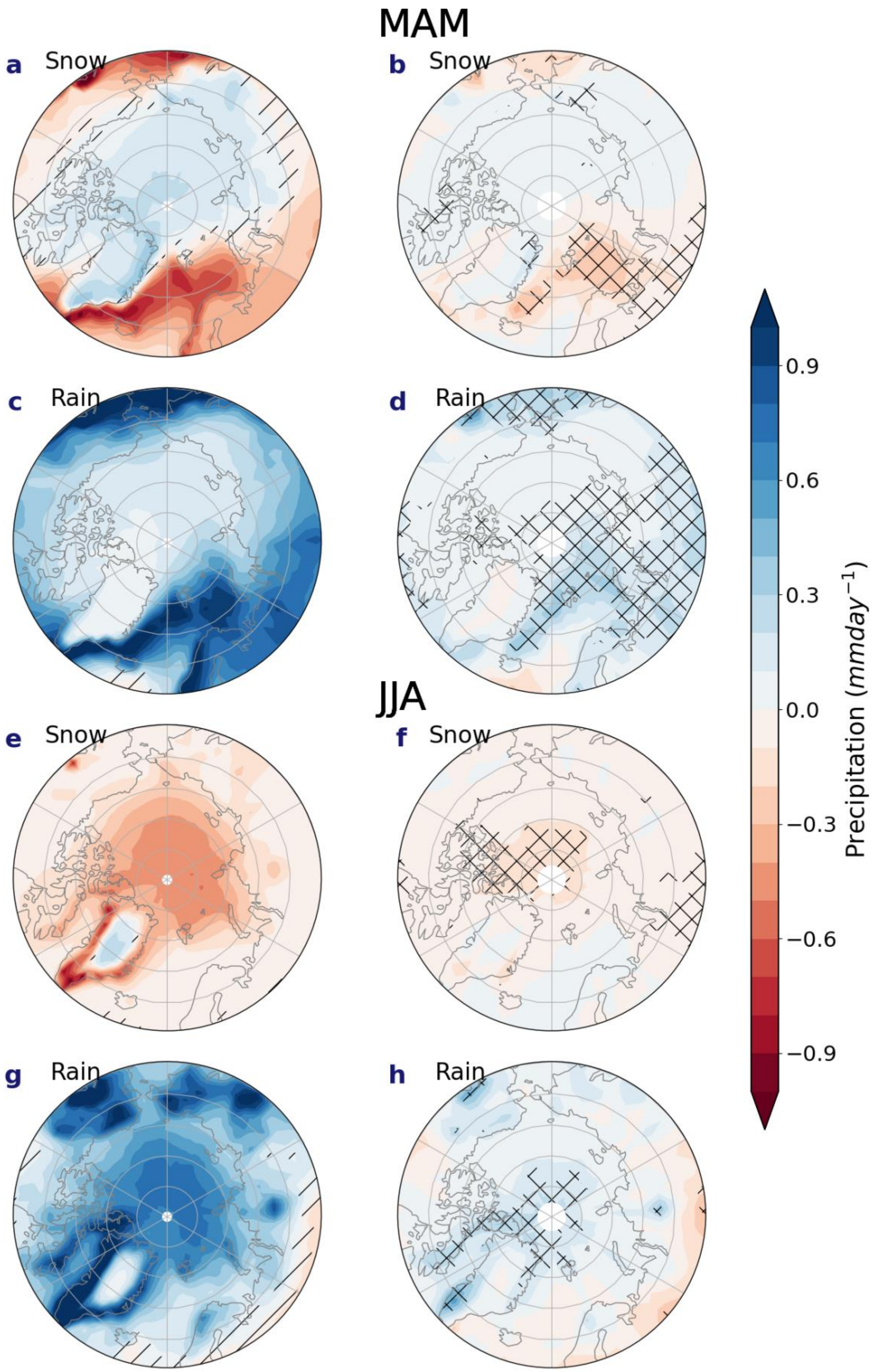
MAM
a Snow
b Snow
c Rain
d Rain
JJA
e Snow
f Snow
g Rain
h Rain
0.9
0.6
0.3
0.0
−0.3
−0.6
−0.9
Precipitation ($mmday^{-1}$)

Supplementary Figure 3: Snowfall and rainfall in CMIP6 and differences between CMIP6 and CMIP5 by the end of the century compared to the start of the century in spring and summer. Left hand column shows the changes in [a,e] snowfall and [c, g] rainfall at the end of the century in March-May (MAM) [a,c] and June-August (JJA) [e,g]. Straight line hatching indicates regions where differences are not statistically significant at the 95% confidence level. Right hand column shows the difference in [b,f] snowfall and [d,h] rainfall at the end of the century (2091-2100) relative to the start of the century (2005-2014) between CMIP5 and CMIP6 (CMIP6-CMIP5) for [b,d] MAM and [f,h] JJA. Dotted hatching indicates statistical significance at 95% confidence level.

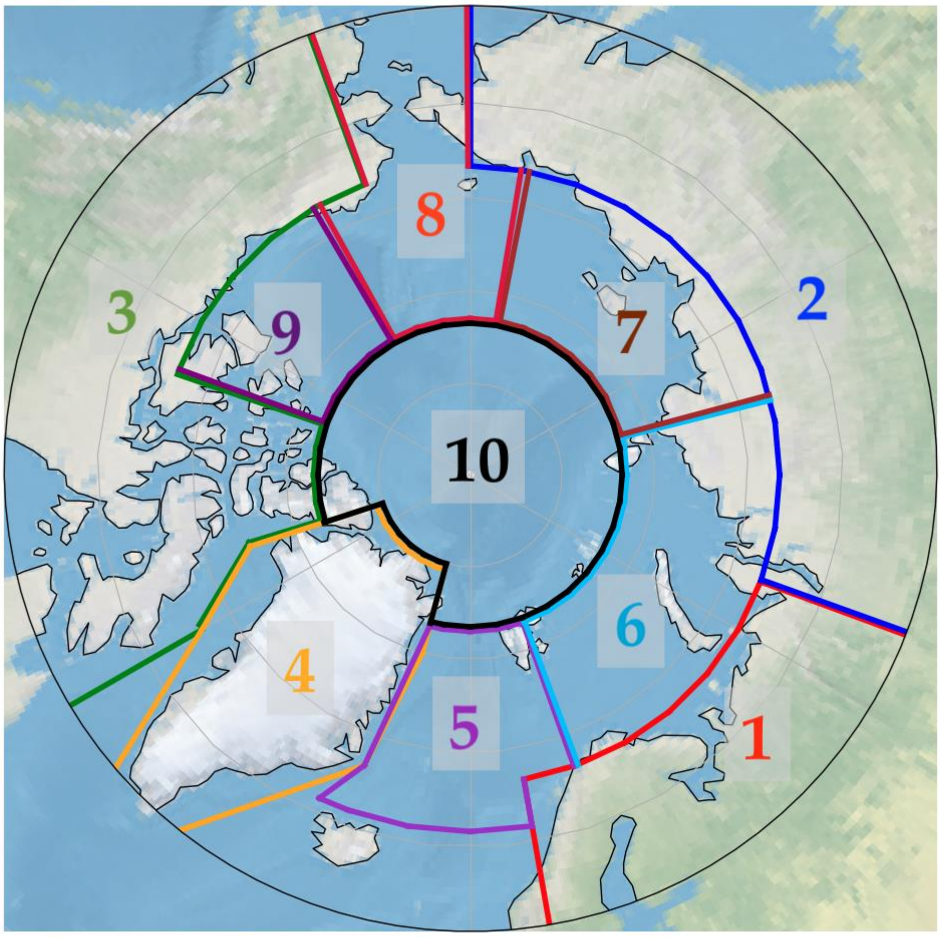

Supplementary Figure 4: Regions of the Arctic. Regions in the Arctic as identified for Figure 9 in manuscript to determine regional snowfall ratio change per 1.5ºC, 2ºC or 3ºC global warming. Regions are identified as [1] West Russia and Europe, [2] Siberia, [3] North America, [4] Greenland, [5] Greenland and Norweigan Seas, [6] Barents and Kara Seas, [7] Laptev and East Siberian Seas, [8] Chukchi and Bering Seas, [9] Beaufort Seas, [10] Central Arctic.

Precipitation type	Season	Experiment	Mean	Standard Deviation	5th Percentile	95th Percentile	5-95th percentile range
**PR (mm day^{-1})**	DJF	CMIP5	0.45	0.15	0.25	0.77	0.53
		CMIP6	0.48	0.19	0.22	0.78	0.56
	MAM	CMIP5	0.27	0.08	0.14	0.42	0.29
		CMIP6	0.28	0.12	0.15	0.49	0.34
	JJA	CMIP5	0.28	0.10	0.14	0.42	0.28
		CMIP6	0.29	0.12	0.13	0.49	0.37
	SON	CMIP5	0.57	0.15	0.35	0.85	0.50
		CMIP6	0.61	0.22	0.36	0.96	0.59
**PRSN (mm day^{-1})**	DJF	CMIP5	0.23	0.07	0.11	0.32	0.21
		CMIP6	0.21	0.08	0.10	0.35	0.26
	MAM	CMIP5	0.05	0.04	0.00	0.13	0.13
		CMIP6	0.03	0.05	-0.05	0.11	0.16
	JJA	CMIP5	-0.23	0.08	-0.42	-0.15	0.27
		CMIP6	-0.26	0.10	-0.42	-0.12	0.30
	SON	CMIP5	-0.16	0.15	-0.45	0.05	0.50
		CMIP6	-0.22	0.18	-0.46	0.08	0.54
**PRRF (mm day^{-1})**	DJF	CMIP5	0.22	0.14	0.05	0.52	0.47
		CMIP6	0.29	0.18	0.07	0.65	0.59
	MAM	CMIP5	0.22	0.10	0.08	0.41	0.33
		CMIP6	0.26	0.12	0.12	0.50	0.39
	JJA	CMIP5	0.52	0.16	0.30	0.80	0.50
		CMIP6	0.56	0.15	0.36	0.80	0.44
	SON	CMIP5	0.73	0.28	0.38	1.26	0.88
		CMIP6	0.85	0.36	0.40	1.41	1.01

Supplementary Table 1: Means, standard deviations and 5th and 95th percentiles of total precipitation (PR), snowfall (PRSN) and rainfall (PRRF) for 2091-2100 for CMIP6 and CMIP5 for DJF (December-February), MAM (March-May), JJA (June-August), SON (September-November), relative to 1981-2009 baseline.

Variable	Season	Experiment	Mean	Standard Deviation	5th Percentile	95th Percentile	5-95th percentile range
**TAS (°C)**	DJF	CMIP5	12.42	2.99	8.75	18.69	9.94
		CMIP6	15.58	4.03	9.94	22.23	12.29
	MAM	CMIP5	7.3	2.25	4.31	11.68	7.37
		CMIP6	9.4	2.89	5.69	14.31	8.62
	JJA	CMIP5	4.02	1.42	2.37	7.09	4.72
		CMIP6	5.66	2.5	2.77	9.85	7.08
	SON	CMIP5	9.43	1.98	5.95	11.77	5.82
		CMIP6	10.61	3.22	7.43	15.74	8.31
**Open Water (million km^2)**	DJF	CMIP5	5753859	2299006	2447948	10131997	7684050
		CMIP6	8553950	2705051	3641489	12029132	8387643
	MAM	CMIP5	4128152	2002527	1394218	8183412	6789194
		CMIP6	6838710	3299192	2662599	11734230	9071632
	JJA	CMIP5	4557727	1226302	2827450	6017898	3190448
		CMIP6	5477015	1336653	3468397	7101497	3633100
	SON	CMIP5	5335868	1594490	3081426	7319145	4237719
		CMIP6	5558909	1630481	2202230	7013214	4810984
**VIMF (kg m^{-1} s^{-1})**	DJF	CMIP5	4.27	1.49	1.86	6.85	4.99
		CMIP6	5.27	2.17	2.26	8.55	6.29
	MAM	CMIP5	4.26	1.65	2.05	7.62	5.57
		CMIP6	4.83	1.73	2.56	7.9	5.34
	JJA	CMIP5	10.92	4.87	3.56	19.16	15.6
		CMIP6	13.26	4.9	5.88	19.4	13.52
	SON	CMIP5	6.51	2.1	3.87	10.5	6.63
		CMIP6	7.24	2.43	4.27	11.65	7.38

Supplementary Table 2: Mean, standard deviation, 5th and 95th Percentiles for surface air temperature (TAS), Open Water and vertically integrated moisture flux (VIMF) for 2091-2100 relative to 1981-2009 baseline in CMIP6 and CMIP5 for DJF (Decemeber-February), MAM (March-May), JJA (June-August), and SON (September-November).

		DJF		MAM		JJA		SON	
		CMIP6	CMIP5	CMIP6	CMIP5	CMIP6	CMIP5	CMIP6	CMIP5
Open Water	Snowfall	-0.21	0.21	-0.31	**-0.36**	-0.61	**-0.59**	-0.34	0.09
	Rainfall	**0.90**	**0.80**	**0.91**	**0.82**	**0.72**	**0.57**	**0.52**	0.18
TAS	Snowfall	0.06	**0.38**	-0.21	-0.26	-0.75	-0.67	**-0.67**	-0.32
	Rainfall	**0.85**	**0.72**	**0.89**	**0.86**	**0.76**	**0.72**	**0.83**	**0.59**
VIMF	Snowfall	-0.36	0.07	**-0.41**	-0.33	**-0.55**	**-0.35**	**-0.63**	**-0.60**
	Rainfall	**0.87**	**0.81**	**0.83**	**0.78**	**0.69**	**0.44**	**0.76**	**0.68**

Supplementary Table 3: Correlations of open water, surface air temperature (TAS) and vertically integrated moisture flux (VIMF) to snowfall and rainfall for each season and across all CMIP6 and CMIP5 models. Numbers in bold indicate statistical significance at 95% confidence level.

Model	Institution	Model Resolution	Variables used
**ACCESS1-0**	Commonwealth Scientific and Industrial Research Organization/Bureau of Meteorology, Australia	1.25º x 1.875º	PR, PRSN, PRRF, TAS, SIC, V, Q
**ACCESS1-3**	Commonwealth Scientific and Industrial Research Organization/Bureau of Meteorology, Australia	1.25º x 1.875º	PR, PRSN, PRRF, TAS, SIC, V, Q
**BCC-CSM1-1**	Beijing Climate Center, China Meteorological Administration	2.8º x 2.8º	PR, PRSN, PRRF, TAS, SIC, V, Q
**BCC-CSM1-1-m**	Beijing Climate Center, China Meteorological Administration	2.8º x 2.8º	PR, PRSN, PRRF, TAS, SIC, V, Q
**BNU-ESM**	Beijing Normal University, China	2.8º x 2.8º	PR, PRSN, PRRF, TAS, SIC, V, Q
**CanESM2**	Canadian Centre for Climate Modelling and Analysis	2.8º x 2.8º	PR, PRSN, PRRF, TAS, SIC,
**CCSM4**	National Center for Atmospheric Research (NCAR), USA	1.0º x 1.25	PR, PRSN, PRRF, TAS, SIC, V, Q
**CESM1-BGC**	National Science Foundation/Department of Energy NCAR, USA	1.0º x 1.25º	PR, PRSN, PRRF, TAS, SIC, V, Q
**CESM1-CAM5**	National Science Foundation/Department of Energy NCAR, USA	1.0º x 1.25º	PR, PRSN, PRRF, TAS, SIC, V, Q
**CMCC-CESM**	Centro Euro-Mediterraneo per i Cambiamenti, Italy	3.4º x 3.75º	PR, PRSN, PRRF, TAS, SIC, V, Q
**CMCC-CM**	Centro Euro-Mediterraneo per i Cambiamenti, Italy	0.75º x 0.75º	PR, PRSN, PRRF, TAS, SIC, V, Q
**CMCC-CMS**	Centro Euro-Mediterraneo per i Cambiamenti, Italy	3.7º x 3.75º	PR, PRSN, PRRF, TAS, SIC, V, Q
**CNRM-CM5**	Centre National de Recherches Meteorologiques, Meteo-France	1.4º x 1.4º	PR, PRSN, PRRF, TAS, SIC, V, Q
**CNRM-CM5-2**	Centre National de Recherches Meteorologiques, Meteo-France	1.4º x 1.4º	V,Q
**CSIRO-Mk3-6-0**	Australian Commonwealth Scientific and Industrial Research Organization	1.9º x 1.9º	PR, PRSN, PRRF, TAS, SIC, V, Q
**FGOALS-g2**	Instute of Atmospheric Physics, Chinese Academy of Sciences, Tsinghua University	2.8º x 2.8º	PR, PRSN, PRRF, TAS, SIC, V, Q
**FIO-ESM**	The First Institude of Oceanography, State Oceanic Administration, China	1.25º x 0.9º	PR, PRSN, PRRF, TAS, SIC, V, Q
**GFDL-CM3**	Geophysical Fluid Dynamics Laboratory, USA	2.0º x 2.5º	PR, PRSN, PRRF, TAS, SIC, V, Q
**GFDL-ESM2G**	Geophysical Fluid Dynamics Laboratory, USA	2.0º x 2.5º	PR, PRSN, PRRF, TAS, SIC, V, Q
**GFDL-ESM2M**	Geophysical Fluid Dynamics Laboratory, USA	2.0º x 2.5º	PR, PRSN, PRRF, TAS, SIC, V, Q
**GISS-E2-H**	Goddard Institute for Space Studies, USA	2.0º x 2.5º	PR, PRSN, PRRF, TAS, SIC, V, Q
**GISS-E2-H-CC**	Goddard Institute for Space Studies, USA	2.0º x 2.5º	PR, PRSN, PRRF, TAS, SIC, V, Q
**GISS-E2-R**	Goddard Institute for Space Studies, USA	2.0º x 2.5º	PR, PRSN, PRRF, TAS, SIC, V, Q
**GISS-E2-R-CC**	Goddard Institute for Space Studies, USA	2.0º x 2.5º	PR, PRSN, PRRF, TAS, SIC, V, Q
**HadGEM2-CC**	UK Met Office Hadley Centre	1.25º x 1.875º	PR, PRSN, PRRF, TAS, SIC
**HadGEM2-ES**	UK Met Office Hadley Centre	1.25º x 1.875º	PR, PRSN, PRRF, TAS, SIC, V, Q

**INMCM4**	Institute for Numerical Mathematics, Russia	1.5º x 2.0º	PR, PRSN, PRRF, TAS, SIC, V, Q
**IPSL-CM5A-LR**	Institut Pierre-Simon Laplace, France	1.9º x 3.75º	PR, PRSN, PRRF, TAS, SIC, V, Q
**IPSL-CM5A-MR**	Institut Pierre-Simon Laplace, France	1.3º x 2.5º	PR, PRSN, PRRF, TAS, SIC, V, Q
**IPSL-CM5B-LR**	Institut Pierre-Simon Laplace, France	1.9º x 3.75º	PR, PRSN, PRRF, TAS, SIC, V, Q
**MIROC-ESM**	Model for Interdisciplinary Research on Climate, Japan	2.8º x 2.8º	PR, PRSN, PRRF, TAS, SIC, V, Q
**MIROC-ESM-CHEM**	Model for Interdisciplinary Research on Climate, Japan	2.8º x 2.8º	PR, PRSN, PRRF, TAS, SIC, V, Q
**MIROC5**	Model for Interdisciplinary Research on Climate, Japan	1.4º x 1.4º	PR, PRSN, PRRF, TAS, SIC, V, Q
**MPI-ESM-LR**	Max Planck Institute for Meteorology, Germany	1.9º x 1.9º	PR, PRSN, PRRF, TAS, SIC, V, Q
**MPI-ESM-MR**	Max Planck Institute for Meteorology, Germany	1.9º x 1.9º	PR, PRSN, PRRF, TAS, SIC, V, Q
**MRI-CGCM3**	Meteorological Research Institute, Japan	1.0º x 1.0º	PR, PRSN, PRRF, TAS, SIC, V, Q
**NorESM1-M**	Norwegian Climate Centre	1.9º x 2.5º	PR, PRSN, PRRF, TAS, SIC, V, Q
**NorESM1-ME**	Norwegian Climate Centre	1.9º x 2.5º	PR, PRSN, PRRF, TAS, SIC, V, Q

Supplementary Table 4: List of all models used from CMIP5 including model resolution and variables used from each model

Model	Institution	Model Resolution	Variables used
**ACCESS-CM2**	Commonwealth Scientific and Industrial Research Organization/Bureau of Meteorology, Australia	1.9˚x 1.3˚	PR, PRSN, PRRF, SIC, TAS, V, Q
**ACCESS-ESM1-5**	Commonwealth Scientific and Industrial Research Organization/Bureau of Meteorology, Australia	1.9˚ x 1.2˚	PR, PRSN, PRRF, SIC, TAS, V, Q
**AWI-CM-1-1-MR**	Alfred Wegnder Institude, Germany	0.9˚ x 0.9˚	PR, PRSN, PRRF, TAS, V, Q
**BCC-CSM2-MR**	Beijing Climate Center, China Meteorological Administration	1.1˚ x 1.1˚	PR, PRSN, PRRF ,SIC, TAS
**CESM2-WACCM**	National Science Foundation/Department of Energy NCAR, USA	1.3˚ x 0.9˚	SIC, TAS, V, Q
**CESM2**	National Science Foundation/Department of Energy NCAR, USA	1.3˚ x 0.9˚	SIC, TAS, V, Q
**CIESM**	Department of Earth System Science/Ministry of Education Key Laboratory for Earth System Modeling, Tsinghua University, Beijing, China	0.9˚ x 1.3˚	SIC, TAS, V, Q
**CMCC-CM2-SR5**	Centro Euro-Mediterraneo per i Cambiamenti, Italy	0.9˚ x 1.3˚	SIC, V, Q
**CNRM-CM6-1**	Centre National de Recherches Meteorologiques, Meteo-France	1.4˚ x 1.4˚	PR, PRSN, PRRF, SIC, TAS, V, Q
**CNRM-CM6-1-HR**	Centre National de Recherches Meteorologiques, Meteo-France	0.5˚x 0.5˚	PR, PRSN, PRRF, SIC, TAS, V, Q
**CNRM-ESM2-1**	Centre National de Recherches Meteorologiques, Meteo-France	1.4˚ x 1.4˚	PR, PRSN, PRRF, SIC, TAS, V, Q
**CanESM5**	Canadian Centre for Climate Modelling and Analysis	2.8˚ x 2.8˚	PR, PRSN, PRRF, SIC, TAS, V, Q
**CanESM5-CanOE**	Canadian Centre for Climate Modelling and Analysis	2.8˚ x 2.8˚	PR, PRSN, PRRF, SIC, TAS, V, Q
**EC-Earth3**	EC-Earth Consortium	0.7˚ x 0.7˚	PR, PRSN, PRRF, SIC, TAS, V, Q
**EC-Earth3-Veg**	EC-Earth Consortium	0.7˚ x 0.7˚	PR, PRSN, PRRF, SIC, TAS, V, Q
**FGOALS-f3-L**	Instute of Atmospheric Physics, Chinese Academy of Sciences, Tsinghua University	1.3˚ x 0.9˚	PR, PRSN, PRRF, SIC, TAS, V, Q
**FGOALS-g3**	Instute of Atmospheric Physics, Chinese Academy of Sciences, Tsinghua University	2 x 2.3˚	PR, PRSN, PRRF, SIC, TAS, V, Q
**FIO-ESM-2-0**	The First Institude of Oceanography, State Oceanic Administration, China	1.3˚ x 0.9˚	SIC, TAS, V, Q
**GFDL-ESM4**	Geophysical Fluid Dynamics Laboratory, USA	1.3˚ x 1˚	PR, PRSN, PRRF, TAS
**GISS-E2-1-G**	Goddard Institute for Space Studies, USA	2˚ x 2.5˚	PR, PRSN, PRRF, TAS, V, Q
**HadGEM3-GC31-LL**	UK Met Office Hadley Centre	1.88˚ x 1.25˚	PR, PRSN, PRRF, SIC, TAS, V, Q
**HadGEM3-GC31-MM**	UK Met Office Hadley Centre	0.83˚ x 0.56˚	PR, PRSN, PRRF, SIC, TAS, V, Q
**INM-CM4-8**	Institute of Numerical Mathematics of the Russian Academy of Sciences, Russia	2˚ x 1.5˚	PR, PRSN, PRRF, TAS, V, Q
**INM-CM5-0**	Institute of Numerical Mathematics of the Russian Academy of Sciences, Russia	2˚ x 1.5˚	PR, PRSN, PRRF, TAS, V, Q
**IPSL-CM6A-LR**	Institut Pierre-Simon Laplace, France	2.5˚ x 1.3˚	PR, PRSN, PRRF, SIC, TAS, V, Q

**KACE-1-0-G**	National Institute of Meteorological Research, Republic of Korea	1.3˚ x 0.9˚	PR, PRSN, PRRF , TAS, V, Q
**MCM-UA-1-0**	Department of Geosciences, University of Arizona	2.2˚ x 3.8˚	PR, PRSN, PRRF, TAS, V, Q
**MIROC-ES2L**	Model for Interdisciplinary Research on Climate, Japan	2.8˚ x 2.8˚	PR, PRSN, PRRF, SIC, TAS, V, Q
**MIROC6**	Model for Interdisciplinary Research on Climate, Japan	1.4˚ x 1.4˚	PR, PRSN, PRRF, SIC, TAS, V, Q
**MPI-ESM1-2-HR**	Max Planck Institute for Meteorology, Germany	0.9˚ x 0.9˚	PR, PRSN, PRRF, SIC, TAS, V, Q
**MPI-ESM1-2-LR**	Max Planck Institute for Meteorology, Germany	1.9˚ x 1.9˚	PR, PRSN, PRRF, SIC, TAS, V, Q
**MRI-ESM2-0**	Meteorological Research Institute, Japan	1.1˚ x 1.1˚	PR, PRSN, PRRF, SIC, TAS, V, Q
**NESM3**	Nanjing University of Information Science and Technology, China	1.9˚ x 1.9˚	PR, PRSN, PRRF, SIC, TAS, V, Q
**NorESM2-LM**	Norwegian Climate Centre	2.5˚ x 1.9˚	PR, PRSN, PRRF, SIC, TAS, V, Q
**NorESM2-MM**	Norwegian Climate Centre	0.9˚ x 1.3˚	PR, PRSN, PRRF, SIC, TAS, V, Q
**UKESM1-0-LL**	UK Met Office Hadley Centre	1.9˚ x 1.3˚	PR, PRSN, PRRF, SIC, TAS, V, Q

Supplementary Table 5: List of all CMIP6 models used including their institution, resolution and variables used in each.
